# Comparative adaptability assessment of bread wheat and synthetic hexaploid genotypes under saline conditions using physiological, biochemical, and genetic indices

**DOI:** 10.3389/fpls.2024.1336571

**Published:** 2024-06-10

**Authors:** Fahad Alghabari, Zahid Hussain Shah

**Affiliations:** ^1^ Department of Arid Land Agriculture, King Abdulaziz University, Jaddah, Saudi Arabia; ^2^ Department of Plant Breeding and Genetics, Pir Mehr Ali Shah Arid Agriculture University, Rawalpindi, Pakistan

**Keywords:** gene expression, salinity stress, antioxidant, photosynthesis, transpiration, PCA

## Abstract

The tolerance to salinity stress is an intricate phenomenon at cellular and whole plant level that requires the knowledge of contributing physiological and biochemical processes and the genetic control of participating traits. In this context, present study was conducted with objective to evaluate the physiological, biochemical, and genetic responses of different wheat genotypes including bread wheat (BW) and synthetic hexaploids (SHs) under saline and control environment. The experiment was conducted in two factorial arrangement in randomized complete block design (RCBD), with genotypes as one factor and treatments as another factor. A significant decline in physiological traits (chlorophyll, photosynthesis, stomatal conductance, transpiration, and cell membrane stability) was observed in all genotypes due to salt stress; however, this decline was higher in BW genotypes as compared to four SH genotypes. In addition, the biochemical traits including enzymes [superoxide dismutase, catalase, and peroxidase (POD)] activity, proline, and glycine betaine (GB) illustrated significant increase along with increase in the expression of corresponding genes (*TaCAT1*, *TaSOD*, *TaPRX2A*, *TaP5CS*, and *TaBADH-A1*) due to salt stress in SHs as compared to BW. Correspondingly, highly overexpressed genes, *TaHKT1;4*, *TaNHX1*, and *TaAKT1* caused a significant decline in Na^+^/K^+^ in SH as compared to BW genotypes under salt stress. Moreover, correlation analysis, principal component analysis (PCA), and heatmap analysis have further confirmed that the association and expression of physiological and biochemical traits varied significantly with salinity stress and type of genotype. Overall, the physiological, biochemical, and genetic evaluation proved SHs as the most useful stock for transferring salinity tolerance to other superior BW cultivars via the right breeding program.

## 1 Introduction

Salinity is a potential constraint drastically affecting approximately 20% of arable land that is increasing dramatically due to unpredictable climate changes and anthropogenic activities (El Sabagh et al., 2020). The continuously increasing human population threatening the world food security in the future as the world food demands will increase up to 70% by 2050 (Bahar et al., 2020). Wheat is the most important cereal crop ranked globally first in context of grain production for human diet (Sendhil et al., 2022). Approximately 36% global population is using wheat as main staple food, providing 55% carbohydrates and 20% calories (Erenstein et al., 2022). Salinity stress results in osmotic stress and ion toxicity in plant cell through perturbing the osmotic balance and by creating ionic imbalance (Shah et al., 2017). This ionic imbalance hinders the uptake of various vital nutrients and their transport to target cells that hamper plant essential processes of plants (Shah et al., 2017; Pastuszak et al., 2022). Furthermore, salinity causes substantial decrease in wheat production owes to poor seedling establishment, reduced plant growth, and hampered plant production (Kizilgeci et al., 2020). Salinity disrupts the ultra-structure of cell components, deteriorates the membrane integrity, damages the photosynthetic machinery, enhances the production of reactive oxygen species (ROS), and declines the catalytic tendency of vital enzymes, which as a whole limit the agronomic productivity of crops (Rajput et al., 2021). Crop growth under saline stress is improved by optimizing the concentrations of osmoprotectants such as proline and glycine betaine (GB), regulating the stomatal conductance, improving the photosynthetic efficiency, and transpiration efficiency, stabilizing the osmotic potential, and by strengthening the antioxidant response of plants (Kesawat et al., 2023). Wheat is highly susceptible to salinity as compared to other field crops. The knowledge of physio-chemical and genetic mechanisms of wheat responses to salinity stress is important to devise a breeding program for developing salinity tolerant wheat verities (Sendhil et al., 2022). Tolerance of plants to salinity is a multigentic traits governed by many genes; therefore, it is important to elucidate the expression of salinity associated genes under saline stress (Omrani et al., 2022). While wheat’s large genome and lack of effective genetic maps have made it difficult to identify many salinity-tolerant genes, the majority of these genes have been found in rice and Arabidopsis (Aycan et al., 2022). In wheat, *TaP5CS* is an important gene regulating proline production and imparting wheat tolerance to salinity (Aycan et al., 2022). In addition, under saline stress, the high expression of *TaKHT1;4* facilitates the efflux of Na^+^ from leaf blades due to high influx of K^+^ (Dave et al., 2022). The membrane transporters, including high-affinity K^+^ transporters (HKTs) and Na^+^ proton exchangers (NHXs) are important proteins that act as carrier of Na^+^ and reduce the Na-toxicity through vacuolar compartmentalization at cellular and whole plant level (Almeida et al., 2017; Assaha et al., 2017). Both *HKTs* and *NHX* are responsible for Na^+^ exclusion and low Na^+^/K^+^ ratio (Almeida et al., 2017). Similarly, AKT1, an inward-rectifying K^+^ channel, first cloned from *Arabidopsis thaliana*, has high selectivity for K^+^ over Na^+^ (Buschmann et al., 2000). In addition, the overexpression of *AKT1* improves plant osmotic stress tolerance by increasing the cellular K^+^ level (Ahmad et al., 2016). Plants are naturally equipped with antioxidant defense mechanisms, which is capable to stop the overproduction of ROS. For instance, increase in the activity of antioxidant enzymes including SOD, POD, and CAT detoxify the ROS such as H_2_O_2_ and O_2_ produced due to inefficient photosynthesis and photorespiration. As explained, GB is an important osmoprotectant that accumulates in plants under various stresses, can reduce the plant osmotic potential, and help the plant to retain water without interfering with vital biochemical processes (Fan et al., 2012; Wang et al., 2014, 2018). Therefore, the enhanced expression of the betaine aldehyde dehydrogenase (*BADH*) in wheat regulates the synthesis of GB under salinity stress (Sun et al., 2019). As salinity simultaneously impacts the physio-chemical and genetic traits of wheat; therefore, these traits should primarily be considered as selection indices while selecting wheat genotypes tolerant against salinity (Shah et al., 2017). Therefore, current study aimed to thoroughly investigate the impacts of salinity stress on physio-chemical and genetic traits of different wheat genotypes. Moreover, to test how the variation of different traits is associated with specific genes in wheat genotypes depicting physiological and biochemical tolerance.

## 2 Materials and methods

### 2.1 Wheat germplasm and experimental site

In present study, the wheat germplasm (**Table 1**) including both bread wheat (BW) and synthetic hexaploids (SHs) was collected from NARC Islamabad, Pakistan, was evaluated for salinity tolerance based on physiological, biochemical, and molecular indices, using two factorial arrangements, with wheat genotypes as one and treatment (control and salinity) as second factor. The tri-replicate pot experiment was performed in RCBD (randomized complete block design) at the research station of King Abdulaziz University, Jeddah, Saudi Arabia, 21°32′36″N and 39°10′22″E, and 12 m above from sea level.

**Table 1:** List of salinity tolerant wheat genotypes used for genes expression analysis.

Genotypes	Pedigree
Bread wheat	
Punjab-11	INQALAB 91*2/TUKURU
Gold-16	FORLANI/ACC//ANA or Fln/ACS//ANA
Zincol-16	SH-88/90A-204//MH97
Shahkar-13	BURGUS/SORT 12–13//KAL/BB/3/PAK 81
Ujala-16	PRL/PASTOR//2236
MA-21	Pb96/Watan/MH-97
Synthetic hexaploid	
SH1	TC870344/GU1//TEMPORALERA M 87/AGR/3/WBLL1
SH2	GPO8 KAZAKSTAN 6 WM98–99/4/KAUZ//ALTAR 84/AOS/3/KAUZ/5//KAUZ//ALTAR 84/AOS/3/KAUZ
SH3	CROC_1/AE.SQUAROSSA (205)//BORL95/3/KENNEDY
SH4	PAM94/3/ALTAR 84/AEGILOPS SQUAROSA(TAUS)//OPATA/PASTOR

### 2.2 Plant growth, treatment, and sampling

The experiment was conducted using pots with size 2.5 L (30 cm depth, 20 cm diameter). Natural soil was used to fill the pots, and each pot was sown with six seeds. At seedling stage, pots were subjected to thinning and four plants per pot were retained. The experimental conditions were optimized in glasshouse at 60% humidity, 25/15 ± 2°C day/night temperature and 10h photoperiod (Al-Ashkar et al., 2021). We irrigated the pots using 500 mL of 25% Hoagland nutrient solution twice a week. After 2 weeks, the experimental set of plants were irrigated with nutrient solution containing 150 mM of NaCl, while the control set of plants were watered in the same way but without NaCl. The salinity stress was started at tillering stage 5 (Feekes growth scale) and kept continued till the appearance of salinity symptoms such as wilting, chlorosis, or conditional leaf rolling and afterward watered in normal way. Plants of control set were watered till optimum level as per requirement. The crop growth was monitored throughout growth season and cultural practices such as hoeing and weeding were applied as per requirement. For each treatment, five pots were used, and for the data of each treatment, five plants were randomly selected in every replicate.

### 2.3 Assessment of biochemical contents

#### 2.3.1 Enzymatic activity

The catalytic activity of antioxidant enzymes was recorded spectrophotometrically following the protocols described by Alici and Arabaci (2016). For the assay of enzymatic activity, the leaf samples of wheat were stored at −20°C and washed thoroughly with distilled water. Subsequently, 10 g of sliced wheat samples were homogenously mixed in 50 mL of 100 mM sodium phosphate buffer having 1 mM ascorbic acid and 0.5% w/v polyvinylpyrrolidone. The homogenate was kept in buffer system for 5 min at 4°C and filtered. To isolate the supernatant, the filtrate followed centrifugation of 5 min at 5000 × g. The catalytic activity of catalase (CAT) was measured using spectrophotometer, by recording reduction in absorbance at 240 nm, at room temperature using H_2_O_2_ as a substrate. In the same way, the activity of peroxidase (POD) was measured using the substrate 4-methylcatechol. For this, the absorption was recorded at 420 nm with the help of spectrophotometer. Finally, the catalytic activity of superoxide dismutase (SOD) was recorded on account of its tendency to stop the photo-reduction of nitroblue-tetrazolium. The SOD reaction was carried out at room temperature by exposing the reaction mixture to the white light for 10–15 min. Following this the spectrophotometric absorption was quantified at 560 nm, and SOD activity was measured in enzyme units.

#### 2.3.2 Glycine betaine and proline

For the estimation of GB, leaves were crushed and homogenized in de-ionized water to isolate the supernatant. Afterwards, the supernatant was completely mixed in 2 N H_2_SO_4_ and KI-I_2_ reagent and subjected to centrifugation at 3000 g for 5 min. Subsequently, dichloro-ethane was added in the mixture, and pink color absorbance was estimated at 365 nm to record the GB in accordance to the method of Grieve and Grattan (1983). Furthermore, the proline was isolated in 3% sulphosalysilic acid, and reactivity of isolate was calculated against acidic-ninhydrin and glacial-acetic acid. Following the reaction mixture was extracted in toluene after heating at 80°C. Subsequently, the proline concentration was recorded by colorimetric method and absorbance was recorded at 520 nm following the method described by Bates et al. (1973).

#### 2.3.3 Estimation of Na^+^/K^+^ ratio

The Na^+^ and K^+^ content from leaf samples were measured following the method opted by Al-Ashkar et al. (2021). Briefly, the oven dried leaf samples were ground to fine powder, and 0.4 g of each sample was dissolved in 3 ml of HClO_4_ and 8 ml of conc HNO_3_ and kept for 12h. Subsequently, the mixture was burned for 3h at 300°C. Afterwards, the distilled water was added to sample till the final volume of 50 mL. Furthermore, the Na^+^ and K^+^ concentration was recorded by a flame photometer (Sigma-Aldrich, St. Louis, Missouri, United States), and Na^+^/K^+^ were calculated.

### 2.4 Assessments of physiological indices

The physiological indices of wheat genotypes such as stomatal-conductance (Gs), photosynthesis-rate (Pn), and transpiration-rate (Tr) were quantified using a portable device IRGA (ADC Bioscientific, Hoddesdon EN11 0NT, UK) from attached flag leaves. The cell membrane stability percentage (CMSP) was measured by estimating electrolyte leakage from leaves following the relative conductivity method (Ibrahim and Quick, 2001). Leaf samples after washing were kept in vials having 10 ml de-ionized water at 10°C for 18h. Afterwards, these vials were kept for one hour at room temperature. Furthermore, samples were subjected to incubation for 24h at 10°C to facilitate the electrolyte diffusion from leaf to water. Further samples were brought back to room temperature and shaken to record electric conductance E1 using Odeon instrument (Aqualabo, Pakistan). Following this, the autoclaving of samples were carried out for 15 min by adjusting temperature at 121°C and pressure at 0.10 MPa to release all electrolytes from plant tissues. The samples were brought to room temperature and shaken to measure electrical conductance E2. Finally, the CMSP was recorded using formula [CMSP = 1−(E1/E2) × 100].

### 2.5 RNA extraction and gene expression

For gene expression, the wheat plants were selected at the onset of salinity symptom. For RNA extraction leaf taken from selected plant samples are instantaneously frozen in liquid nitrogen and stored at −80°C till the start of procedure. The RNA extraction was carried out using RNeasy kit (Qiagen, Germany) following the instruction given. Total RNA of 2 µg was used to construct cDNA library. For this purpose, QuantiTect reverse transcription kit (Qiagen, Germany) was used. To check the transcript level of salinity related genes *TaHKT1;4*, *TaCAT1*, *TaSOD*, *TaPRX2A*, *TaNHX1*, *TaAKT1TaP5CS*, and *TaBADH-A1* (reference provided in **Table 2**) in leaf tissues, the qRT-PCR was performed by a SYBR Green Kit (BioFACT, Korea), and the gene expression was normalized using TaActin1-expressing gene. For all qRT-PCR analysis, each expression profile was analyzed and confirmed using three technical and three biological replicates as used by Ahmed et al. (2022). In addition, to calculate the relative gene expression for each sample double delta Ct value was used (Ahmed et al., 2022). The list of gene primers used for qRT-PCR is given in **Table 2**.

**Table 2:** List of primers used in gene expression analysis.

Gene	Primer sequence	Reference
*TaCAT1*	TCTCTCGGCCAGAAGCTCG (F) AGGGAAGAACTTGGACGGC (R)	Al-Ashkar et al., 2021
*TaNHX1*	TGACGGAGGCAGAAGACCG (F) CCCAAAACTCTACACAGCGT (R)	Al-Ashkar et al., 2021
*TaHKT1;4*	AGCAAGCTGAAGTTGAGGGG (F) AGAGTTGTGACAGAGCCGTG (R)	Dave et al., 2022
*TaP5CS*	GAAGGCTCTTATGGGTGTACTCAA (F) TAAAAGACCTTCAACACCCACAGG (R)	Aycan et al., 2022
*TaSOD*	TCCTTTGACTGGCCCTAATG (F) CTTCCACCAGCATTTCCAGT (R)	Jiang et al., 2019
*TaPRX2A*	CGTGTGTGTGATCATCAGTAAC (F) AGGCGGAGTGTAAATTACAAGA (R)	Su et al., 2020
*TaBADH-A1*	ACTCCCCGTTAATATCCCAGTCT (F) AGAGCCTGGTTTTGGAAGTCTTT (R)	Yu et al., 2022
*TaAKT1*	CGGATAATGCCGTGAATG (F) TTATACTATCCTCCATGCCT (R)	Zeeshan et al., 2020

### 2.6 Data analysis

The Analysis of variance (ANOVA) was performed using a computer-based software Statistix ver. 8.1 (McGraw-Hill, 2008), at the probability level of 5%. Moreover, RStudio version 1.1.456 (RStudio Team, 2020) was used to construct correlation chart, principal component analysis (PCA) biplot and the heatmap clusters. The R packages “factoextra” and “FactoMineR” were used to establish the PCA biplot, while the R packages “GGally” and “ggplot2” were used to execute Pearson’s correlation. Furthermore, the heatmap cluster dendrogram was constructed using the R packages, “pheatmap” and “complex Heatmap”.

## 3 Results

### 3.1 Biochemical and physiological traits

**Figure 1:**
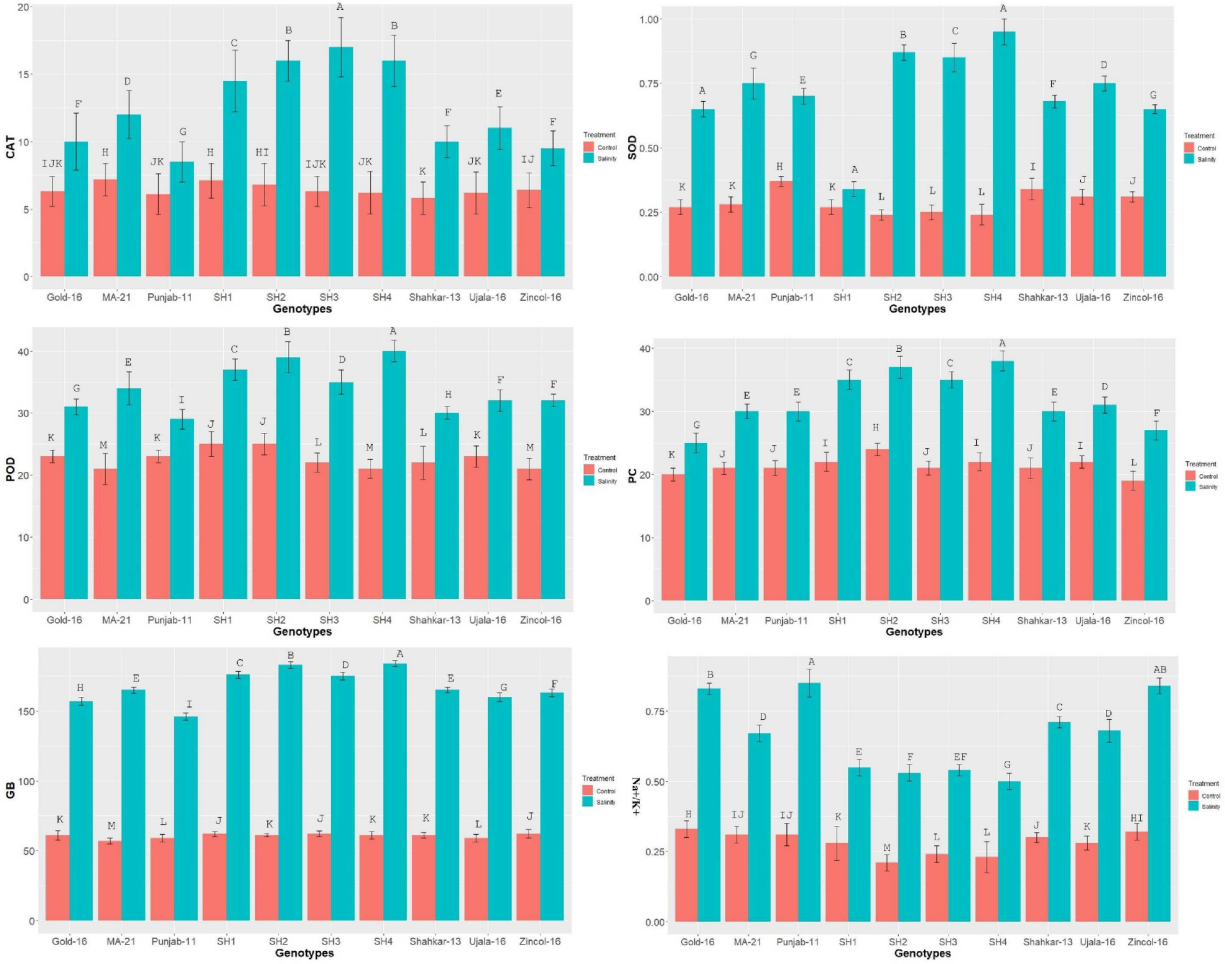
The effect of control and saline conditions on the activities of antioxidant enzymes and concentrations of proline and glycine betaine (GB) in different bread wheat and synthetic hexaploid (SH) wheat genotypes. The data indicated in figure represent the mean values averaged and analyzed on the appearance of salinity symptoms during tri-replicate experiment at *p* ≤ 0.05. *The bar values having different letters vary significantly at *p* ≤ 0.05. Units: SOD, POD and POX activity (U mg^−1^ of protein); Proline Content (μgg-1FW); Glycine betaine (μgg^−1^ FW).

The catalytic activity of the antioxidant enzymes POD, CAT, and SOD varied significantly in all wheat genotypes under saline condition as compared to control treatment (**Figure 1**). The enzymatic activity in terms of enzymatic unit (U mg^-1^ protein) increased more dramatically in SH genotypes as compared to bread wheat (BW) genotypes. Among BW genotypes, MA-21 and Zincol-16 illustrated a significantly high increase in the enzymatic activity due to salinity compared to other BW genotypes. In parallel to the enzymatic activity, the proline and GB contents were also increased in both SH and BW genotypes due to salt treatment as indicated in **Figure 1**. In general, SH wheat genotypes showed significantly higher enzymatic activity, and proline and GB content under saline condition as compared to BW cultivars. All wheat genotypes including SHs illustrated significant rise in Na^+^/K^+^ ratio under salinity as compared to control condition (**Figure 1**). The SH genotypes followed by MA-21, Ujala-16 and Shahkar-13 illustrated least dramatic rise in Na^+^/K^+^ ratio due to high influx of K^+^, however the BW genotypes Gold-16, Punjab-11 and Zincol-15 showed more dramatic rise in Na^+^/K^+^ probably due to less influx of K^+^.

**Figure 2:**
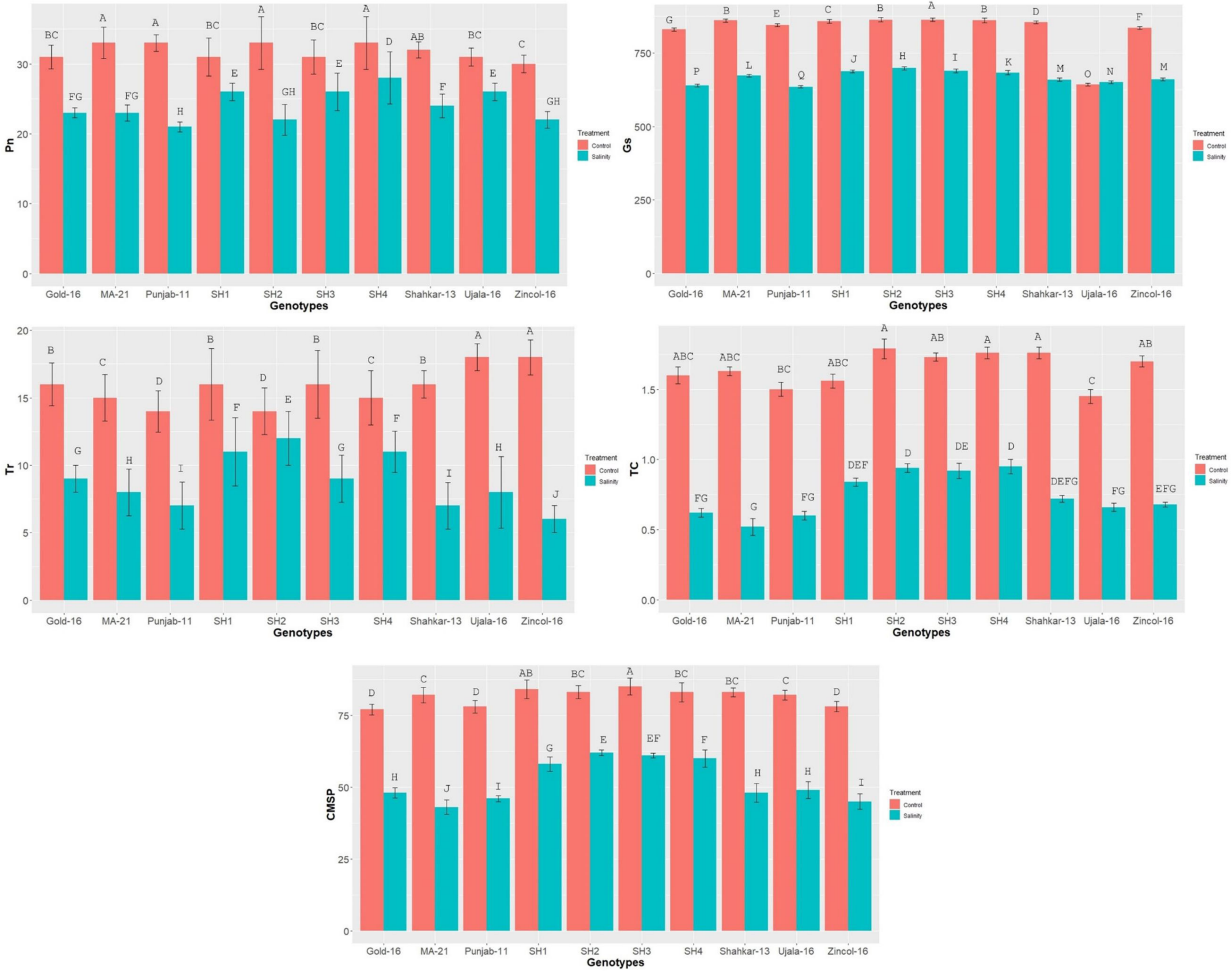
The effect of control and saline conditions on the physiological traits of different bread wheat and synthetic hexaploid (SH) wheat genotypes. The data indicated in figure represent the mean values averaged and analyzed on the appearance of salinity symptoms during tri-replicate experiment at *p* ≤ 0.05. *The bar values having different letters vary significantly at *p* ≤ 0.05. Units: Pn (μmm^−1^S^−2^); Tr (mmm^−1^S^−2^); Gs(mmm^−1^S^−2^); Chlorophyll (gkg^−1^).

The physiological indices such as stomatal-conductance (Gs), total chlorophyll (chl), transpiration-rate (Tr), photosynthesis-rate (Pn), and CMSP exhibited significant reduction in all wheat genotypes due to saline condition as compared to control application (**Figure 2**). The physiological traits illustrated high variation among BW cultivars as compared to SH wheat genotypes due to salinity) treatment. Among BW cultivars, Ujala and Zincol-16 showed significantly higher mean values of physiological traits as compared to other BW cultivars.

### 3.2 Statistical evaluations

**Figure 3:**
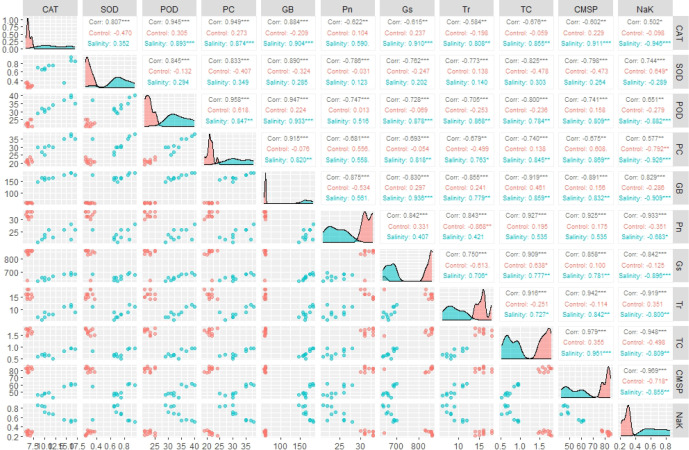
Correlogram illustrating the effect of control and salinity treatment on paired association of physiological and biochemical traits. *** = Significant at *p* ≤ 0.001; ** = Significant at *p* ≤ 0.01. (CAT, catalase), (SOD, superoxide dismutase), (POD, peroxidase), (PC, proline content), (GB, glycine betaine), (Pn, photosynthesis rate), (Gs, stomatal conductance), (Tr, transpiration rate), (TC, total chlorophyll), (CMSP, cell membrane stability percentage).

Correlogram has illustrated varying values of correlation coefficient among physiological and biochemical traits, in both positive and negative direction. Overall, the traits did not show significant association under control conditions; however, they depicted more significant and strong paired association under saline condition (**Figure 3**). Among traits, photosynthesis (Pn) showed a statistically significant correlation with stomatal conductance (Gs) and total chlorophyll content. In addition, all antioxidant enzymes including SOD, POD and POD showed a significantly high paired association in positive direction, among them and with GB and proline content. On the other hand, CMSP demonstrated positive paired association with the SOD, POD, CAT, Pn, Gs, and total chlorophyll content. Conversely, the transpiration rate (Tr) depicted significantly high association in negative direction with Pn and Gs as shown in **Figure 3**. In addition, the correlation analysis proved that all traits are strongly associated and change as a unit under salt stress.

**Figure 4:**
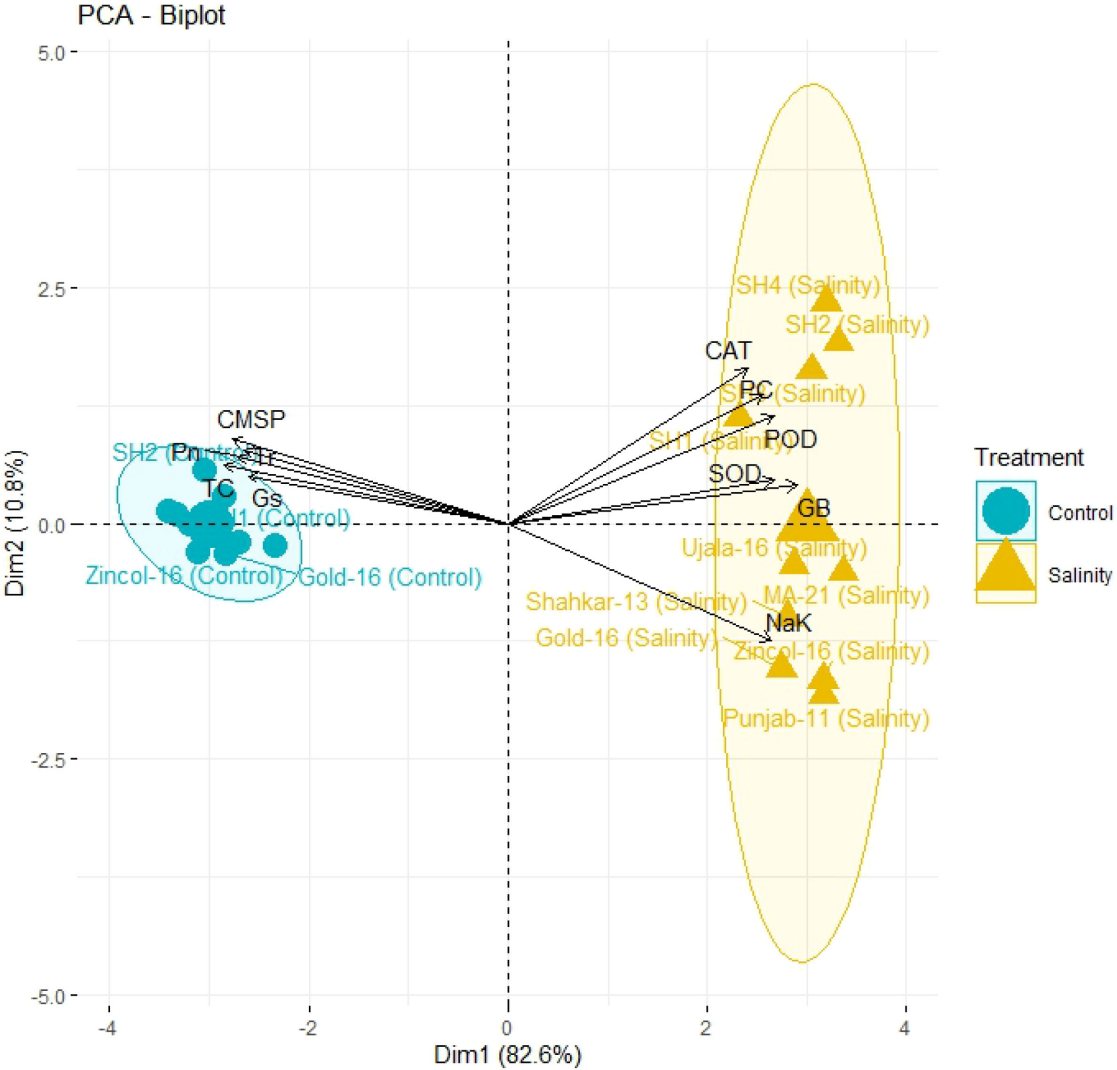
The principal component analysis (PCA) based upon correlation matrix, indicates the impact of salt and control treatment on the correlation of physiological and biochemical traits in different wheat genotypes. All biochemical traits (SOD, POD, CAT, GB, PC, and Na/K) depicted more close association under salt stress while physiological traits (Pn, Gs, Tr, TC, and CMSP) depicted more strong correlation under control treatment as indicated by their closeness to respective eclipses. The more closely placed genotypes to the trait vectors have more strong expression of traits. (CAT, catalase), (SOD, superoxide dismutase), (POD, peroxidase), (PC, proline content), (GB, glycine betaine), (Pn, photosynthesis rate), (Gs, stomatal conductance), (Tr, transpiration rate), (TC, total chlorophyll), (CMSP, cell membrane stability percentage).

**Figure 5:**
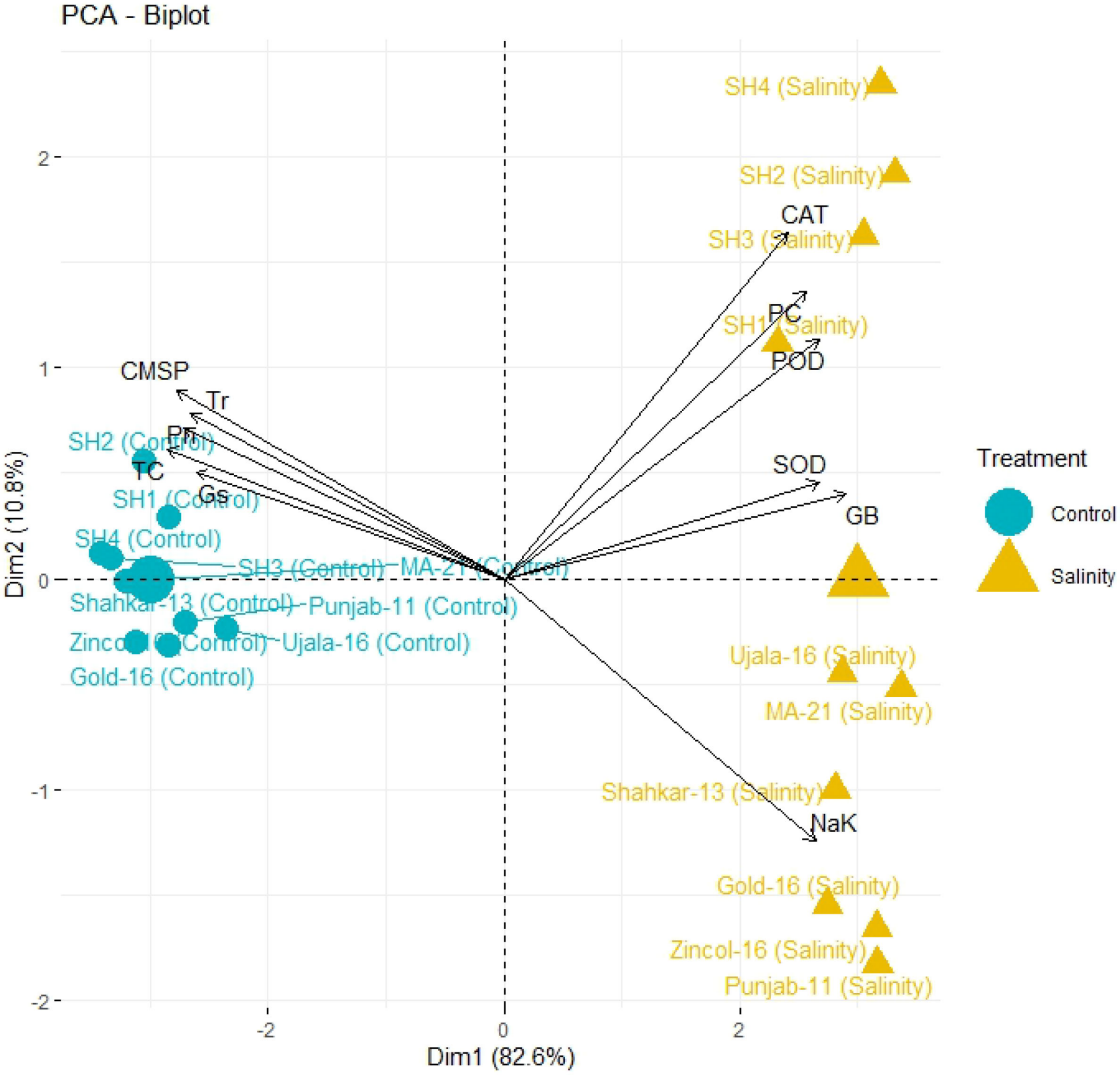
PCA biplot categorizing genotypes into different quadrants based on the trait’s association under control and salinity stress condition. The more closely placed genotypes to the trait vectors have more strong association of traits. (CAT, catalase), (SOD, superoxide dismutase), (POD, peroxidase), (PC, proline content), (GB, glycine betaine), (Pn, photosynthesis rate), (Gs, stomatal conductance), (Tr, transpiration rate), (TC, total 608 chlorophyll), (CMSP, cell membrane stability percentage).

**Figure 6:**
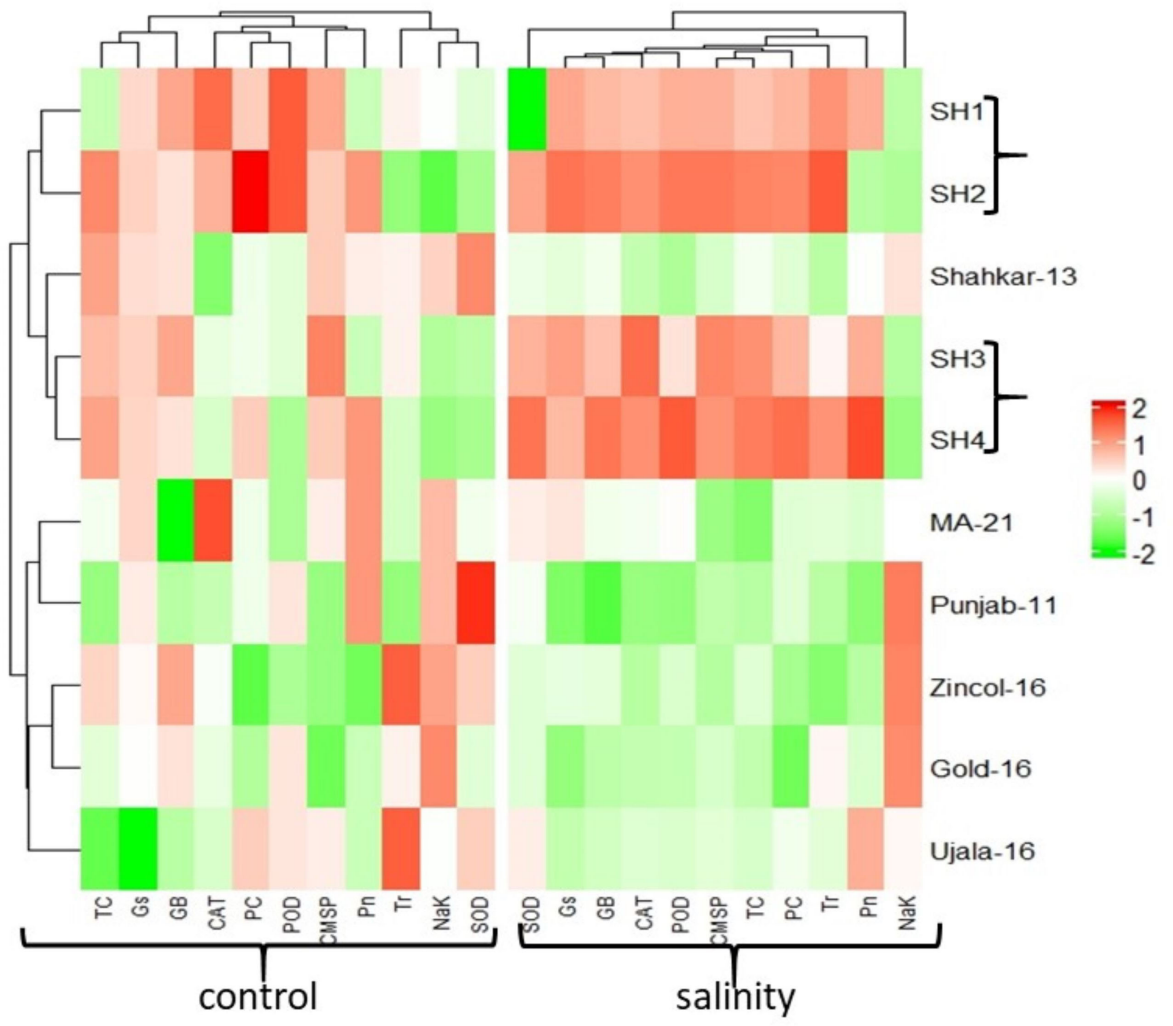
Heatmap analysis among genotypes and traits for the expression of physiological and biochemical traits under control and salinity stress treatment. The varying color pattern of bands (light to dark) indicates the extent by which the expression of traits in each wheat genotype. *White color indicates no effect, color variation from light green to dark green indicates low to high decline in trait expression, and color variation from light red to dark red indicates low to high rise in trait expression. (CAT, catalase), (SOD, 611 superoxide dismutase), (POD, peroxidase), (PC, proline content), (GB, glycine betaine), (Pn, 612 photosynthesis rate), (Gs, stomatal conductance), (Tr, transpiration rate), (TC, total 613 chlorophyll), (CMSP, cell membrane stability percentage).

The PCA biplot also revealed varying impacts of control and salt treatments on the extent of expression, and strength of association of physiological and biochemical traits in both BW and SH wheat genotypes (**Figure 4**). The varying orientation of eclipses in biplot under saline and control conditions confirmed the variation in association and expression of traits owes to changing treatments in all wheat genotypes. On the other hand, PCA biplot for wheat genotypes under saline conditions showed the vectors representing physiological and biochemical traits in more proximity as compared to control conditions (**Figure 5**). Furthermore, under control conditions the BW cultivars (Punjab-11, Gold-16, Zincol-16, Shahkar-13, Ujala-16, MA-21) responded differently as compared to SH wheat genotypes in context of trait association as shown by their disperse distribution in PCA biplot. However, under saline conditions BW genotypes were more closely spaced in one quadrant of PCA biplot, while SH genotypes were more closely spaced in other quadrant of PCA biplot. This was a confirmation of distinct genetic makeup of BW and SH wheat genotypes. Moreover, heatmap cluster analysis further confirmed the varying expression and extent of association of traits in wheat genotypes under salt stress as compared to control treatment (**Figure 6**). The heatmap cluster dendrogram has further rectified the results obtained from PCA. The different bands pattern and clusters distribution in heatmap explicated that the expression and correlation of physiological and biochemical traits is highly dependent upon the type of treatment. In addition, the similar bands pattern for SH genotypes under salinity stress indicates the similarity of traits expression in SH genotypes. In general, all the traits except Na^+^/K^+^ were highly expressed in SH genotypes.

### 3.3 Genetic regulation

**Figure 7:**
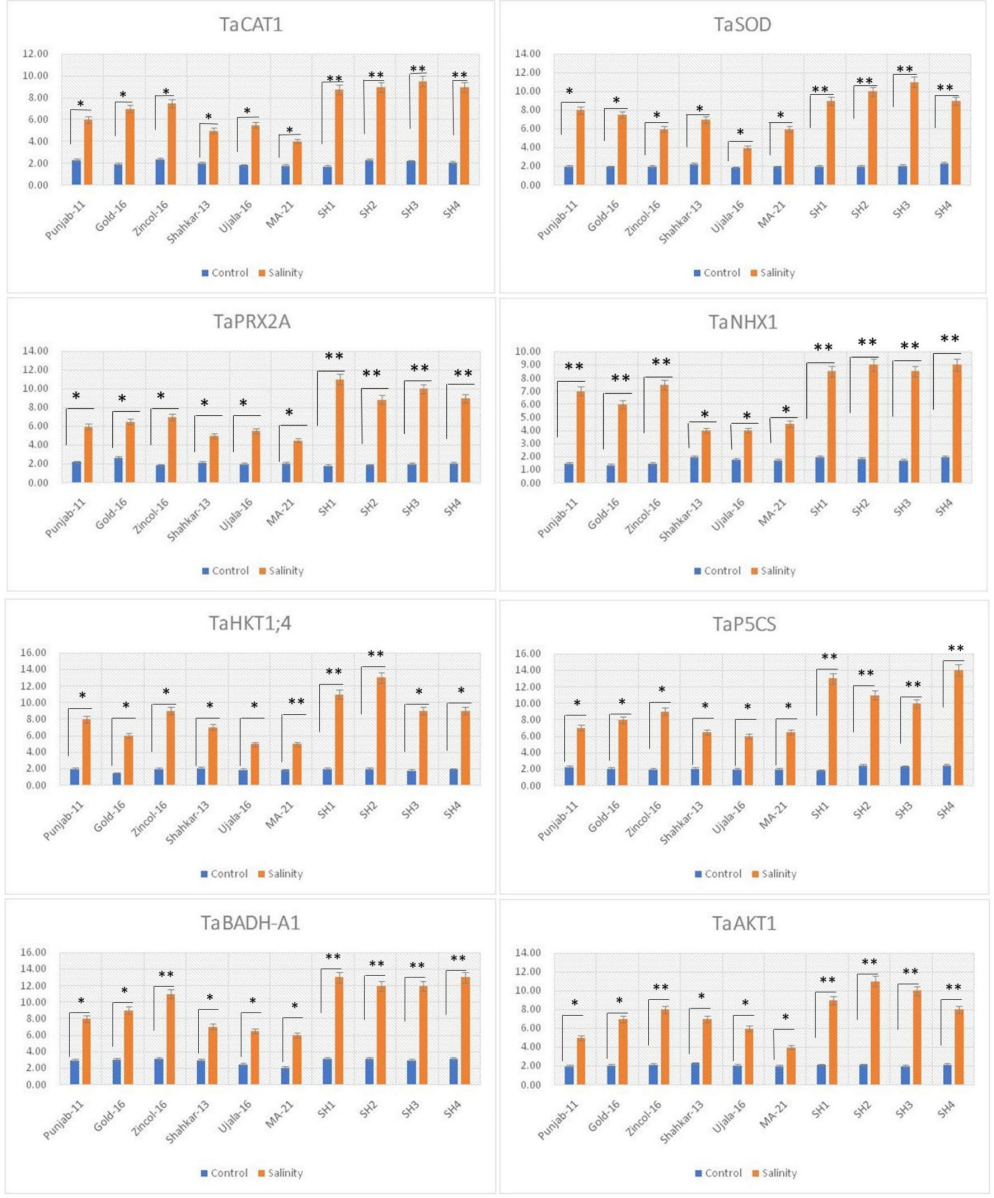
Relative expression analysis of salinity related genes in bread wheat and synthetic hexaploid wheat genotypes under control and salinity treatments. * indicates significance at p ≤ 0.05. , ** indicates significance at p ≤ 0.01.

The genes *TaCAT1, TaSOD* and *TaPRX2A* significantly (*p* ≤ 0.05) upregulated under saline conditions in all wheat genotypes as compared to control treatment, with comparatively high in all SHs (SH1-SH4) wheat lines followed by BW cultivars Zincol-16, Gold-16, and Punjab-11 (**Figure 7**). In addition, the high transcript levels of genes *TaCAT1*, *TaSOD*, and *TaPRX2A* was consistent with the increased catalytic activity of antioxidant enzymes CAT, SOD, and POD in wheat genotypes under high salt conditions. In the same way the genes *TaHKT1;4*, *TaNHX1* and *TaAKT1*, regulating the compartmentalization of K^+^ and Na^+^ ions, were significantly (*p* ≤ 0.05) overexpressed in all wheat genotypes under saline condition as compared to control treatments (**Figure 7**). Correspondingly the SHs (SH1-SH4) followed by Zincol-16 illustrated the significantly (*p* ≤ 0.05) high upregulation of *TaHKT1;4*, *TaNHX1* and *TaAKT1*, with high K^+^-influx and low Na^+^-efflux, as confirmed by the low Na^+^/K^+^ ratio of these genotypes (**Figure 7**). Besides under saline stress, *TaP5CS* depicted maximum increase of expression in SH (SH1-SH4) followed by Zincol-16, while minimum increase of expression in MA-21 (**Figure 7**). Moreover, in all these genotypes, the increase in transcripts level of *TaP5CS* was consistent with increase in the concentration of proline. Likewise, the gene *TaBADH-A1* regulating the synthesis of GB was significantly (p ≤ 0.05) overexpressed in all wheat genotypes under salt stress as compared to control treatment. Correspondingly SH (SH1-SH4) followed by Zincol-16 depicted maximum increase in the expression *TaBADH-A1*, similar to increase in their GB content. Correspondingly, the gene *TaHKT1;4* depicted significant (*p* ≤ 0.05) upregulation in all wheat genotypes due to salinity stress with maximum upregulation in SHs, Gold-16, Punjab-11 and Shahkar-13 and minimum upregulation in MA-21 (**Figure 7**). In general, all wheat genotypes manifested significant (*p* ≤ 0.05) difference in the expression of all stress related gene under saline conditions as compared to control treatments.

## 4 Discussion

In present study elite BW cultivars and SH genotypes were evaluated based upon physiological, biochemical and genetic indices under salt and control environment. Salinity stress creates partially or wholly oxidative stress in plant by enhancing the concentration of ROS, which disrupts redox homeostasis (Shah et al., 2017). To counteract the negative effects of ROS, plants are naturally equipped with various tolerant mechanisms. Plants do not act passively, but rather react actively through the regulation of various mechanisms (Hasanuzzaman et al., 2020). The catalytic activities of antioxidant enzymes enhance in the tolerant wheat genotypes during stress, which could potentially prevent the production of ROS through triggering the scavenging mechanism (Rajput et al., 2021). Salinity stress in wheat genotypes triggers the catalytic-activity of antioxidant enzymes, as reviewed by Shah et al. (2017). Correspondingly current study recorded a substantial increase in the activities of POD, CAT and SOD in all wheat genotypes due to salinity stress (**Figure 1**). The variations in wheat genotypes’ genetic architecture and gene regulation mechanisms are reflected in the varying catalysis potential of antioxidant enzymes (Amro et al., 2022). From this angle, SH wheat lines, which are infused with multiple genetic networks that confer salinity tolerance, showed relatively higher enzymatic activities than BW cultivars (Yang et al., 2014). Correspondingly, in present study the SHs (SH1-SH4) depicted significantly high increase in the activities of enzymes SOD, POD and CAT (**Figure 1**). Abiotic stresses disrupt various physio-chemical processes, which directly impact the metabolic activities of plants (Shah et al., 2017). According to Fahad et al. (2017), drought and salinity stress, for instance, have the propensity to damage the elements of the plant photosystem and the enzymes involved in chlorophyll biosynthesis, which results in a considerable drop in chlorophyll (chl) content and photosynthesis-rate (Pn). Moreover, lipid-peroxidation and protein-degradation caused by salinity stress deteriorate the structural integrity of the membrane and increase the generation of ROS, which disrupt membrane fluidity and cause high electrolyte leakage (Sachdev et al., 2021). In addition, abiotic stress like salinity alters the osmotic potential and water balance of plants, interfering with a number of exchange processes like transpiration (Tr) and stomatal conductance (Gs) (Yang et al., 2021). Accordingly, the present study found a considerable decrease in CMSP, Pn, Gs, Tr, and chlorophyll under saline condition; however, the SHs (SH1-SH4) illustrated comparatively less decrease in these traits that is an indicator of their higher physiological tolerance (**Figure 2**). Qaseem et al. (2019); Alghabari et al. (2021) and Pastuszak et al. (2022) confirmed our findings by observing significantly less decrease in chlorophyll, Pn, Gs, Tr, and CMSP as a result of various abiotic stresses in tolerant wheat genotypes. Furthermore, plants naturally produce a variety of osmolytes, including proline and GB, which help the plant to withstand the harmful effects of abiotic stresses and maintain their osmotic balance (Wang et al., 2010; Qaseem et al., 2019). According to these results, the current study found that salinity stress caused a dramatic increase in the proline and GB content in all wheat genotypes (**Figure 1**). Because all biological processes in plants are highly organized and interconnected, disruption of one biochemical process directly affects another physiological process, and vice versa (Pastuszak et al., 2022) as rectified by correlation analysis (**Figure 3**). Plants experience perturbations in their physio-chemical equilibrium due to abiotic stresses like salinity. According to this viewpoint, a plant’s reaction varies based on its genotype (Kizilgeci et al., 2020). Accordingly, the nature of stress and the types of genotypes were found to have varying effects on the expression and correlation of traits (**Figures 4** **–** **6**). Proline functions as an efficient osmolyte, antioxidant defense, and signaling molecule in response to environmental stressors (Hayat et al., 2012). Proline builds up in stressed plants and functions as an antioxidant to reduce ROS, stabilize membranes to stop electrolyte leakage, and support cell osmotic balance (Kesawat et al., 2023). In the glutamate pathway, 1-pyrroline-5-carboxylate synthase (P5CS) converts glutamate to proline (Sabbioni et al., 2021). As a result, Aycan et al. (2022) discovered that moderate and salt-tolerant genotypes significantly increased the *TaP5CS* gene’s transcript level and proline content when exposed to salt stress. In consistent with these findings, current study recorded dramatic increase in the transcripts level of *TaP5CS* gene in SHs (SH1-SH4) and Zincol-16 exhibiting high salt tolerance based upon physiological and biochemical evaluation (**Figure 7**). The genes responsible for maintaining the equilibrium of Na^+^ and K^+^ ions in wheat under salt stress, known as high-affinity potassium transporters (HKTs), are the most frequently utilized genes in wheat (Kumar et al., 2017). In order for plants to tolerate salt stress, the HKT family of proteins is thought to be crucial (Ali et al., 2019; Zeeshan et al., 2020).

In contrast to the other HKT-type transporter groups, *TaHKT1;4* helps to exclude Na+ from leaf blades in response to salt stress and has a greater functional variety among its members (Dave et al., 2022). In parallel with these findings, present study reported high manifested relatively high expression of *TaHKT1;4* in SHs (SH1-SH4), Zincol-16 and Punjab-11 exhibiting high physiological and biochemical tolerance to salt stress as compared to other genotypes (**Figure 7**). The high expression of *TaCAT1* gene encoding CAT protein detoxifies ROS, particularly H_2_O_2_ in plants subjected to salt stress. Therefore, Al-Ashkar et al. (2021) found approximately twofold increase in the expression of *TaCAT1* gene in wheat genotypes under salt stress conditions as compared to genotypes that exhibited less upregulation of this gene. In complementary with these findings current study reported comparatively high upregulation of *TaCAT1* gene in SHs (SH1-SH4) and BW genotype Zincol-16 showing high salt tolerance based upon physiological and biochemical indices (**Figure 7**). Detoxification of ROS and less cytosolic Na^+^/K^+^ ratio are two key factors that facilitate plants to tolerate salinity (Al-Ashkar et al., 2019). In this context plant CAT proteins are mandatory in H_2_O_2_ scavenging, while Na transporters HKT1 and NHX1 are two fundamental elements in Na^+^ compartmentalization (El-Hendawy et al., 2017). Furthermore, Zeeshan et al. (2020) elucidated the role of inward-rectifying K^+^ channel, *TaAKT1*, whose over-expression remarkably enhanced the influx of K^+^ in wheat genotypes under salt stress to balance the cytosolic Na^+^/K^+^ ratio. Correspondingly, the qPCR analysis revealed that SHs (SH1-SH4) and Zincol-16 depicted higher transcripts level of *TaNHX1* and *TaKHT1;4*, *TaAKT1* along with high expression level of *TaCAT1* (**Figure 7**). The SODs are first line of defense against abiotic stresses in plants (Tyagi et al., 2017). Therefore, enhanced activities of SOD, along with CAT and POD is mandatory to keep balance the concentration of ROS in plants. Jiang et al. (2019) found the enhanced expression of *TaSODs* in wheat genotypes under salt stress, imparts tolerance to oxidative stress. Likewise, Su et al. (2020), analyzed the expression profile of *TaPRX-2A* and the activity of POD protein in transgenic wheat lines under salt stress using qRT-PCR, and concluded that overexpression of *TaPRX-2A* is responsible for high POD activity in transgenic wheat lines exhibiting salt tolerance based upon physiological and biochemical traits. Correspondingly, present study reported a dynamic increase in the activity of POD protein along with higher upregulation of *TaPRX2A* gene in SH (SH1-SH4) and Zincol genotypes exhibiting higher salt tolerance based upon physiological and biochemical evaluation (**Figure 7**). Similarly, Yu et al. (2022), confirmed the role of overexpressed *TaBADH-A1* gene in enhancing GB content in wheat genotypes showing tolerance to salt stress. Likewise, present study further endorsed their findings by recording higher GB content in synthetic hexploids (SH1-SH4), Zincol-16 and Gold-16 genotypes along with higher transcript levels of *TaBADH-A1* (**Figure 7**).

Plant responds against stress like all other living organism at cellular level. The abiotic stress such as salinity perturbs plants physiological and biochemical processes by inducing various oxidative changes within the cellular system (Shah et al., 2017). Plant react to these changes through regulating the expression of genes that sustain osmotic balance and redox homeostasis for maintaining the normal physiological and biochemical processes. Aside from this, all wheat genotypes under the same degree of saline stress showed notable differences in the relative expression of these genes. This served as a sign of the distinct genetic makeup they inherited from their lineage. It is noteworthy that there was a complementary difference in the genetic and biochemical behavior of SHs (SH1-SH4) and Zincol-16 compared to other BW genotypes. This is explained by the greater genetic diversity that they inherited from their closed wild ancestors, which, according to a review by Bhatta et al. (2018), modifies the expression pattern of the genes mentioned above in unison. Overall, in present study the SHs (SH1-SH4) genotypes exhibited high tolerance to salinity based upon physiological and biochemical indices as compared to BW varieties. In present study all SH genotypes manifested the high relative expression of genes the genes increasing proline accumulation, antioxidant enzymes activities, K^+^ influx and Na^+^ efflux under saline conditions. The high expressivity in SHs under salt stress is probably associated with diversity enriched D genome (Yang et al., 2014). SH wheat is a source of bringing the alien genes from wild progenitors for extending the narrow genetic base of local elite BW cultivars. The hexaploid wheat (AABBDD) is more tolerant to abiotic stresses as compared to its tetraploid counterpart (AABB) (Rosyara et al., 2019). It is believed that the hexaploid wheat has attained this tolerance from the wild relative *Aegilops tauschii*, a source of D genome having the high genetic diversity. Perhaps the high salinity tolerance of SHs is due to diverse genes of D genome that needs further exploration based on the integration of genetic traits with their physiological and biochemical indices. In addition, Gold-16, Punjab-11, and Zincol-16 genotypes of BW showed elevated activity of antioxidant enzymes in response to salinity stress, along with high expression of genes associated with salinity, indicating a strong resistance to salinity stress (Shah et al., 2017). Overall, the current study demonstrated that salinity-tolerant genes from the BW cultivars Gold-16, Zincol-16, and Punjab-11, combined with SH wheat genotypes, can be a useful stock for transferring salinity tolerance to other superior BW cultivars via the right breeding program.

## Data availability statement

The original contributions presented in the study are included in the article/supplementary material. Further inquiries can be directed to the corresponding author.

## Author contributions

ZS: Writing – review & editing. FA: Writing – review & editing.
